# Professional Overwork

**Published:** 1878-04

**Authors:** 


Professional Overwork.—Dr. T. A. Emmet, in his admirable memoir of Dr. E. R. Peaslee, read before the Medical Society of the County of New York, remarked that the subject of his sketch died from overwork. No one can doubt this fact, and no one similarly situated as was Dr. Peaslee should fail to heed the lesson which his untimely end affords. It is a well established fact that ultimate success in our profession means hard work, and when this success is attained, it is still harder to restrain a desire to work within proper limits. There is a fascination about professional progress which increases with the means of its gratification until in some it becomes almost irresistible. This fact was strikingly exemplified in the case of Dr. Peaslee. The members of the Medical Society of the County of New York will remember that it was but a short time since that he, in some remarks upon the death of the lamented Dr. A. B. Crosby, referred to the necessity of conserving the vital forces, and guarding against the very excesses of labor to which he himself fell a victim. Every one who heard him on that occasion must have been impressed with the conviction that he appreciated the full force of his remarks and governed himself according to their spirit. But alas, scarcely six months elapsed when, in the same hall, Dr. Peaslee's memoir offers a melancholy verification of his own warnings.—*Med. Record, Feb.* 9, 1878.

